# Supplementary material for: Coarse woody debris decomposition assessment tool: Model development and sensitivity analysis
Source: PLoS One. 2021 Jun 4;16(6):e0251893. doi: 10.1371/journal.pone.0251893 (PMC8177548; doi:10.1371/journal.pone.0251893)
Supplement: S1 Table — *: Lat, latitude; Lon, longitude; all sites are located in North America; the climatic data were downloaded from Daymet database (Thornton et Al., 2016). Annual mean temperature, annual precipitation and elevation at each location are in S2 Table. (DOCX) [file pone.0251893.s001.docx]

S1 Table. Coordinates of eighty-nine sites used to obtain climate data for analyzing model sensitivity*.

| Site | Lat (°) | Lon (°) | Site | Lat (°) | Lon (°) | Site | Lat (°) | Lon (°) |
| --- | --- | --- | --- | --- | --- | --- | --- | --- |
| S01 | 33.15 | -79.8 | S31 | 17 | -91 | S61 | 45 | -85 |
| S02 | 16 | -96 | S32 | 21 | -99 | S62 | 45 | -76 |
| S03 | 17 | -96 | S33 | 21 | -90 | S63 | 50 | -123 |
| S04 | 18 | -97 | S34 | 27 | -81 | S64 | 50 | -113 |
| S05 | 19 | -98 | S35 | 29 | -109 | S65 | 50 | -96 |
| S06 | 20 | -99 | S36 | 29 | -97 | S66 | 50 | -86 |
| S07 | 21 | -99 | S37 | 31 | -91 | S67 | 52.5 | -58 |
| S08 | 22 | -100 | S38 | 35 | -119 | S68 | 55 | -128 |
| S09 | 23 | -105 | S39 | 35 | -79 | S69 | 55 | -116 |
| S10 | 25 | -105 | S40 | 39 | -123 | S70 | 55 | -96 |
| S11 | 27 | -105 | S41 | 39 | -83 | S71 | 55 | -86 |
| S12 | 29 | -105 | S42 | 39 | -76 | S72 | 55 | -76 |
| S13 | 31 | -105 | S43 | 43 | -123 | S73 | 55 | -66 |
| S14 | 33 | -105 | S44 | 43 | -73 | S74 | 60 | -134 |
| S15 | 35 | -105 | S45 | 45 | -69 | S75 | 60 | -125 |
| S16 | 37 | -105 | S46 | 47 | -123 | S76 | 60 | -115 |
| S17 | 39 | -105 | S47 | 47 | -67 | S77 | 60 | -96 |
| S18 | 41 | -105 | S48 | 49 | -77 | S78 | 60 | -76 |
| S19 | 43 | -105 | S49 | 49 | -75 | S79 | 65 | -139 |
| S20 | 45 | -105 | S50 | 51 | -65 | S80 | 65 | -130 |
| S21 | 47 | -105 | S51 | 31.5 | -85 | S81 | 65 | -118 |
| S22 | 49 | -105 | S52 | 35 | -111 | S82 | 65 | -97.5 |
| S23 | 51 | -105 | S53 | 35 | -96 | S83 | 65 | -89 |
| S24 | 53 | -105 | S54 | 35 | -86 | S84 | 14.2 | -90.2 |
| S25 | 55 | -105 | S55 | 40 | -115 | S85 | 14.5 | -91.5 |
| S26 | 57 | -105 | S56 | 40 | -96 | S86 | 15 | -91 |
| S27 | 59 | -105 | S57 | 40 | -86 | S87 | 15.8 | -92.8 |
| S28 | 61 | -105 | S58 | 45 | -118 | S88 | 16.3 | -88.7 |
| S29 | 63 | -105 | S59 | 45 | -110 | S89 | 17.7 | -88.7 |
| S30 | 65 | -105 | S60 | 45 | -96 |  |  |  |

*Lat, latitude; Lon, longitude; all sites are located in North America; the climatic data were downloaded from Daymet database (Thornton et Al., 2016). Annual mean temperature, annual precipitation and elevation at each location are in S2 Table.
