# Supplementary material for: Coarse woody debris decomposition assessment tool: Model development and sensitivity analysis
Source: PLoS One. 2021 Jun 4;16(6):e0251893. doi: 10.1371/journal.pone.0251893 (PMC8177548; doi:10.1371/journal.pone.0251893)
Supplement: S2 Table — * Ele, elevation; meanT, mean air temperature. (DOCX) [file pone.0251893.s002.docx]

S2 Table. Altitude and climate at the sites used for model sensitivity analysis*.

| Site | Ele (m) | meanT  (°C) | Precipitation (mm) | Snowfall (kg m^-2^) | Site | Ele (m) | meanT (°C) | Precipitation (mm) | Snowfall (kg m^-2^) |
| --- | --- | --- | --- | --- | --- | --- | --- | --- | --- |
| S01 | 8 | 18.5 | 1425.7 | 0.74 | S31 | 164 | 26.1 | 2399.6 | 0 |
| S02 | 454 | 25.2 | 882.6 | 0 | S32 | 635 | 22.2 | 1139.3 | 0.11 |
| S03 | 1819 | 16.9 | 1429.6 | 0 | S33 | 3 | 26.3 | 772.5 | 0 |
| S04 | 1046 | 22.0 | 888.0 | 0 | S34 | 6 | 23.1 | 1294.5 | 0 |
| S05 | 2482 | 14.6 | 871.4 | 0 | S35 | 1308 | 18.3 | 710.2 | 0 |
| S06 | 2417 | 15.1 | 492.7 | 0 | S36 | 56 | 21.3 | 1085.8 | 0 |
| S07 | 635 | 22.1 | 1145.9 | 0.11 | S37 | 100 | 18.9 | 1747.2 | 2.32 |
| S08 | 1004 | 21.2 | 449.7 | 0 | S38 | 524 | 16.8 | 252.7 | 0 |
| S09 | 744 | 22.7 | 1204.2 | 0 | S39 | 46 | 16.6 | 1233.4 | 5.16 |
| S10 | 1974 | 16.4 | 505.2 | 0 | S40 | 822 | 13.1 | 1396.1 | 3.05 |
| S11 | 1429 | 18.8 | 333.5 | 0 | S41 | 201 | 11.9 | 1085.7 | 46.95 |
| S12 | 1155 | 19.3 | 289.2 | 0 | S42 | 23 | 13.7 | 1202.9 | 23.58 |
| S13 | 1299 | 16.8 | 263.3 | 2.05 | S43 | 581 | 11.5 | 1338.1 | 4.21 |
| S14 | 1551 | 13.9 | 388.9 | 18.38 | S44 | 665 | 5.0 | 1652.9 | 359.5 |
| S15 | 1627 | 12.8 | 379.0 | 27.79 | S45 | 95 | 6.2 | 1169.1 | 216.65 |
| S16 | 2773 | 5.0 | 582.0 | 232.67 | S46 | 59 | 10.8 | 1525.8 | 2.63 |
| S17 | 2824 | 3.2 | 651.6 | 245.32 | S47 | 558 | 2.5 | 1443.4 | 435.61 |
| S18 | 1951 | 7.4 | 426.9 | 80.55 | S48 | 283 | 1.2 | 868.8 | 209.7 |
| S19 | 1453 | 8.0 | 343.1 | 49.73 | S49 | 474 | 0.2 | 1106.4 | 302.86 |
| S20 | 1207 | 7.3 | 426.5 | 65.84 | S50 | 764 | -2.7 | 1563.5 | 611.94 |
| S21 | 789 | 7.1 | 356.4 | 38.38 | S51 | 99 | 18.8 | 1381.7 | 0.32 |
| S22 | 727 | 4.6 | 364.8 | 53.73 | S52 | 1690 | 12.0 | 295.8 | 14.32 |
| S23 | 535 | 3.2 | 405.7 | 67.57 | S53 | 234 | 16.6 | 1121.2 | 14.95 |
| S24 | 450 | 1.6 | 422.1 | 76.33 | S54 | 401 | 14.4 | 1622.1 | 23.05 |
| S25 | 360 | 0.4 | 461.5 | 109.85 | S55 | 1918 | 8.1 | 316.9 | 65.37 |
| S26 | 519 | -2.2 | 532.5 | 141.31 | S56 | 351 | 11.7 | 871.6 | 47.27 |
| S27 | 357 | -3.5 | 458.8 | 129.85 | S57 | 250 | 11.0 | 1089 | 63.16 |
| S28 | 390 | -6.2 | 412.9 | 135.04 | S58 | 1036 | 8.2 | 399.1 | 42.74 |
| S29 | 256 | -7.6 | 225.0 | 83.90 | S59 | 2293 | 2.4 | 727.9 | 288.86 |
| S30 | 245 | -9.6 | 181.3 | 73.30 | S60 | 317 | 7.3 | 659.2 | 70.11 |

** Ele, elevation; meanT, mean air temperature. Continued

S2 Table. Continued.

| Site | Ele (m) | meanT  (°C) | Precipitation  (mm) | Snowfall  (kg m^-2^) | Site | Ele (m) | meanT (°C) | Precipitation  (mm) | Snowfall  (kg m^-2^) |
| --- | --- | --- | --- | --- | --- | --- | --- | --- | --- |
| S61 | 279 | 6.9 | 888.9 | 142.01 | S76 | 304 | -2.1 | 374.4 | 122.96 |
| S62 | 139 | 6.5 | 971.8 | 180.96 | S77 | 165 | -7.1 | 410.8 | 133.48 |
| S63 | 1547 | 2.8 | 2052.3 | 445.93 | S78 | 107 | -6.7 | 500.8 | 192.01 |
| S64 | 1014 | 5.1 | 424.8 | 78.64 | S79 | 1068 | -7.1 | 586 | 225.17 |
| S65 | 270 | 2.9 | 612.5 | 106.85 | S80 | 1282 | -8.6 | 679.4 | 394.77 |
| S66 | 287 | 0.7 | 719.5 | 181.28 | S81 | 187 | -6.5 | 244.4 | 98.85 |
| S67 | 399 | -1.0 | 1527.3 | 610.63 | S82 | 96 | -10.8 | 234.9 | 95.80 |
| S68 | 1578 | 0.2 | 1499.5 | 448.77 | S83 | 246 | -11.8 | 320.7 | 148.54 |
| S69 | 772 | 2.2 | 529.7 | 120.85 | S84 | 801 | 23.9 | 6322.8 | 25.60 |
| S70 | 234 | -1.6 | 513.6 | 121.59 | S85 | 266 | 26.5 | 5395.8 | 0 |
| S71 | 73 | -2.8 | 426 | 117.27 | S86 | 1889 | 15.4 | 4496.1 | 145.40 |
| S72 | 323 | -4.3 | 874 | 285.81 | S87 | 801 | 24.1 | 2038.2 | 0 |
| S73 | 554 | -4.5 | 851.7 | 314.65 | S88 | 22 | 26.3 | 1633.8 | 0 |
| S74 | 1064 | -2.4 | 576.1 | 260.65 | S89 | 10 | 26.3 | 1447.4 | 0 |
| S75 | 1272 | -4.1 | 771 | 277.28 |  |  |  |  |  |
