# Supplementary material for: Coarse woody debris decomposition assessment tool: Model development and sensitivity analysis
Source: PLoS One. 2021 Jun 4;16(6):e0251893. doi: 10.1371/journal.pone.0251893 (PMC8177548; doi:10.1371/journal.pone.0251893)
Supplement: S3 Table — Values for k1 calculated using Eqs 20 and 21 for downed and standing CWD, respectively; k2, fitted without forcing the intercept; k3 values fitted using forcing the intercept to 100% of the initial mass; and k4 and k5 values were based on Eq 24, respectively. (DOCX) [file pone.0251893.s003.docx]

S3 Table. CWD decomposition constants calculated using different decomposition models for eighty-nine sites. Values for *k*_1_ calculated using Eqn. 20 and Eqn. 21 for downed and standing CWD, respectively; *k*_2_, fitted without forcing the intercept; *k*_3_ values fitted using forcing the intercept to 100% of the initial mass; and *k*_4_ and *k*_5_ values were based on Eqn. 24, respectively.

| Site | Downed CWD | | | | | Standing CWD | | | | | Lat  (°N) | Lon  (°W) |
| --- | --- | --- | --- | --- | --- | --- | --- | --- | --- | --- | --- | --- |
|  | *k*_1_ | *k*_2_ | *k*_3_ | *k*_4_ | *k*_5_ | *k*_1_ | *k*_2_ | *k*_3_ | *k*_4_ | *k*_5_ |  |  |
| S01 | 0.190 | 0.199 | 0.197 | 0.215 | 0.181 | 0.149 | 0.190 | 0.173 | 0.142 | 0.170 | 33.15 | 79.79 |
| S02 | 0.166 | 0.174 | 0.172 | 0.175 | 0.115 | 0.128 | 0.165 | 0.149 | 0.117 | 0.129 | 16.0 | 96.0 |
| S03 | 0.183 | 0.191 | 0.189 | 0.193 | 0.132 | 0.140 | 0.181 | 0.163 | 0.119 | 0.123 | 18.0 | 96.0 |
| S04 | 0.173 | 0.181 | 0.179 | 0.192 | 0.175 | 0.131 | 0.170 | 0.153 | 0.118 | 0.138 | 19.0 | 97.0 |
| S05 | 0.149 | 0.160 | 0.157 | 0.163 | 0.151 | 0.115 | 0.149 | 0.135 | 0.114 | 0.144 | 20.0 | 98.0 |
| S06 | 0.113 | 0.123 | 0.119 | 0.125 | 0.113 | 0.084 | 0.110 | 0.098 | 0.079 | 0.080 | 20.0 | 99.0 |
| S07 | 0.183 | 0.188 | 0.187 | 0.216 | 0.180 | 0.142 | 0.179 | 0.163 | 0.146 | 0.189 | 21.0 | 99.0 |
| S08 | 0.142 | 0.147 | 0.146 | 0.148 | 0.085 | 0.097 | 0.132 | 0.116 | 0.094 | 0.111 | 22.0 | 100.0 |
| S09 | 0.180 | 0.187 | 0.186 | 0.187 | 0.112 | 0.140 | 0.178 | 0.162 | 0.133 | 0.151 | 23.0 | 105.0 |
| S10 | 0.105 | 0.113 | 0.110 | 0.114 | 0.093 | 0.080 | 0.102 | 0.092 | 0.087 | 0.106 | 25.0 | 105.0 |
| S11 | 0.091 | 0.097 | 0.095 | 0.099 | 0.067 | 0.062 | 0.082 | 0.073 | 0.063 | 0.064 | 27.0 | 105.0 |
| S12 | 0.082 | 0.087 | 0.085 | 0.088 | 0.057 | 0.053 | 0.070 | 0.062 | 0.060 | 0.086 | 29.0 | 105.0 |
| S13 | 0.056 | 0.062 | 0.060 | 0.064 | 0.068 | 0.040 | 0.050 | 0.045 | 0.047 | 0.066 | 31.0 | 105.0 |
| S14 | 0.062 | 0.068 | 0.066 | 0.070 | 0.069 | 0.049 | 0.060 | 0.055 | 0.055 | 0.066 | 33.0 | 105.0 |
| S15 | 0.050 | 0.056 | 0.054 | 0.057 | 0.057 | 0.042 | 0.050 | 0.046 | 0.049 | 0.075 | 35.0 | 105.0 |
| S16 | 0.051 | 0.055 | 0.054 | 0.056 | 0.048 | 0.042 | 0.049 | 0.046 | 0.048 | 0.052 | 37.0 | 105.0 |
| S17 | 0.049 | 0.055 | 0.053 | 0.057 | 0.063 | 0.041 | 0.049 | 0.045 | 0.047 | 0.063 | 39.0 | 105.0 |
| S18 | 0.037 | 0.041 | 0.040 | 0.043 | 0.052 | 0.032 | 0.038 | 0.035 | 0.039 | 0.059 | 41.0 | 105.0 |
| S19 | 0.029 | 0.033 | 0.031 | 0.034 | 0.041 | 0.027 | 0.031 | 0.029 | 0.032 | 0.046 | 43.0 | 105.0 |
| S20 | 0.039 | 0.045 | 0.043 | 0.047 | 0.073 | 0.035 | 0.042 | 0.039 | 0.041 | 0.060 | 45.0 | 105.0 |
| S21 | 0.036 | 0.042 | 0.039 | 0.044 | 0.072 | 0.032 | 0.038 | 0.036 | 0.039 | 0.065 | 47.0 | 105.0 |
| S22 | 0.037 | 0.043 | 0.041 | 0.045 | 0.075 | 0.033 | 0.040 | 0.037 | 0.040 | 0.062 | 49.0 | 105.0 |
| S23 | 0.048 | 0.054 | 0.052 | 0.056 | 0.065 | 0.042 | 0.050 | 0.046 | 0.049 | 0.067 | 51.0 | 105.0 |
| S24 | 0.054 | 0.060 | 0.058 | 0.062 | 0.063 | 0.047 | 0.056 | 0.052 | 0.055 | 0.075 | 53.0 | 105.0 |
| S25 | 0.060 | 0.066 | 0.064 | 0.068 | 0.065 | 0.052 | 0.062 | 0.058 | 0.061 | 0.086 | 55.0 | 105.0 |
| S26 | 0.050 | 0.056 | 0.054 | 0.057 | 0.053 | 0.044 | 0.051 | 0.048 | 0.051 | 0.071 | 57.0 | 105.0 |
| S27 | 0.048 | 0.053 | 0.051 | 0.054 | 0.048 | 0.041 | 0.048 | 0.045 | 0.048 | 0.065 | 59.0 | 105.0 |
| S28 | 0.034 | 0.037 | 0.036 | 0.038 | 0.033 | 0.030 | 0.034 | 0.032 | 0.035 | 0.043 | 61.0 | 105.0 |
| S29 | 0.018 | 0.020 | 0.019 | 0.021 | 0.031 | 0.017 | 0.020 | 0.019 | 0.020 | 0.024 | 63.0 | 105.0 |
| S30 | 0.009 | 0.011 | 0.010 | 0.012 | 0.032 | 0.010 | 0.013 | 0.012 | 0.014 | 0.034 | 65.0 | 105.0 |
| S31 | 0.193 | 0.199 | 0.199 | 0.210 | 0.165 | 0.159 | 0.193 | 0.180 | 0.166 | 0.211 | 17.0 | 91.0 |
| S32 | 0.183 | 0.188 | 0.187 | 0.198 | 0.140 | 0.142 | 0.178 | 0.163 | 0.143 | 0.182 | 21.0 | 99.0 |
| S33 | 0.156 | 0.164 | 0.162 | 0.165 | 0.099 | 0.118 | 0.154 | 0.138 | 0.109 | 0.112 | 21.0 | 90.0 |
| S34 | 0.192 | 0.200 | 0.199 | 0.203 | 0.158 | 0.153 | 0.192 | 0.176 | 0.136 | 0.143 | 27.0 | 81.0 |

Continued

S3 Table. Continued

| S35 | 0.128 | 0.134 | 0.132 | 0.136 | 0.092 | 0.101 | 0.125 | 0.115 | 0.105 | 0.119 | 29.0 | 109.0 |
| --- | --- | --- | --- | --- | --- | --- | --- | --- | --- | --- | --- | --- |
| S36 | 0.176 | 0.185 | 0.183 | 0.185 | 0.135 | 0.137 | 0.175 | 0.159 | 0.122 | 0.132 | 29.0 | 97.0 |
| S37 | 0.191 | 0.201 | 0.199 | 0.203 | 0.158 | 0.152 | 0.193 | 0.176 | 0.133 | 0.141 | 31.0 | 91.0 |
| S38 | 0.072 | 0.078 | 0.076 | 0.080 | 0.062 | 0.046 | 0.061 | 0.054 | 0.052 | 0.077 | 35.0 | 119.0 |
| S39 | 0.181 | 0.191 | 0.189 | 0.193 | 0.150 | 0.142 | 0.181 | 0.164 | 0.135 | 0.162 | 35.0 | 79.0 |
| S40 | 0.133 | 0.144 | 0.141 | 0.146 | 0.141 | 0.112 | 0.138 | 0.127 | 0.117 | 0.143 | 39.0 | 123.0 |
| S41 | 0.132 | 0.141 | 0.138 | 0.143 | 0.126 | 0.112 | 0.135 | 0.126 | 0.117 | 0.130 | 39.0 | 83.0 |
| S42 | 0.145 | 0.154 | 0.152 | 0.155 | 0.123 | 0.121 | 0.147 | 0.137 | 0.126 | 0.147 | 39.0 | 76.0 |
| S43 | 0.129 | 0.139 | 0.136 | 0.141 | 0.139 | 0.110 | 0.133 | 0.124 | 0.112 | 0.120 | 43.0 | 123.0 |
| S44 | 0.107 | 0.116 | 0.114 | 0.118 | 0.111 | 0.092 | 0.111 | 0.103 | 0.105 | 0.148 | 43.0 | 73.0 |
| S45 | 0.122 | 0.131 | 0.128 | 0.132 | 0.116 | 0.105 | 0.125 | 0.117 | 0.113 | 0.132 | 45.0 | 69.0 |
| S46 | 0.132 | 0.141 | 0.139 | 0.143 | 0.125 | 0.113 | 0.136 | 0.126 | 0.124 | 0.162 | 47.0 | 123.0 |
| S47 | 0.096 | 0.104 | 0.102 | 0.106 | 0.101 | 0.083 | 0.100 | 0.093 | 0.093 | 0.114 | 47.0 | 67.0 |
| S48 | 0.090 | 0.098 | 0.095 | 0.099 | 0.084 | 0.078 | 0.093 | 0.087 | 0.087 | 0.099 | 49.0 | 77.0 |
| S49 | 0.087 | 0.095 | 0.092 | 0.097 | 0.085 | 0.075 | 0.090 | 0.084 | 0.084 | 0.097 | 49.0 | 75.0 |
| S50 | 0.082 | 0.090 | 0.087 | 0.092 | 0.086 | 0.069 | 0.084 | 0.078 | 0.077 | 0.092 | 51.0 | 65.0 |
| S51 | 0.187 | 0.197 | 0.195 | 0.199 | 0.154 | 0.147 | 0.188 | 0.171 | 0.142 | 0.173 | 31.5 | 85.0 |
| S52 | 0.038 | 0.040 | 0.039 | 0.041 | 0.031 | 0.032 | 0.035 | 0.034 | 0.034 | 0.022 | 35.0 | 111.0 |
| S53 | 0.161 | 0.168 | 0.166 | 0.168 | 0.113 | 0.128 | 0.159 | 0.146 | 0.130 | 0.154 | 35.0 | 96.0 |
| S54 | 0.156 | 0.165 | 0.163 | 0.166 | 0.127 | 0.129 | 0.157 | 0.146 | 0.129 | 0.137 | 35.0 | 86.0 |
| S55 | 0.026 | 0.029 | 0.028 | 0.029 | 0.032 | 0.024 | 0.027 | 0.026 | 0.027 | 0.033 | 40.0 | 115.0 |
| S56 | 0.112 | 0.119 | 0.117 | 0.121 | 0.090 | 0.095 | 0.114 | 0.106 | 0.101 | 0.104 | 40.0 | 96.0 |
| S57 | 0.125 | 0.134 | 0.131 | 0.135 | 0.117 | 0.108 | 0.128 | 0.120 | 0.116 | 0.139 | 40.0 | 86.0 |
| S58 | 0.039 | 0.043 | 0.041 | 0.044 | 0.051 | 0.034 | 0.039 | 0.037 | 0.039 | 0.040 | 45.0 | 118.0 |
| S59 | 0.050 | 0.055 | 0.053 | 0.057 | 0.060 | 0.041 | 0.050 | 0.046 | 0.048 | 0.060 | 45.0 | 110.0 |
| S60 | 0.091 | 0.099 | 0.097 | 0.104 | 0.097 | 0.078 | 0.094 | 0.087 | 0.089 | 0.120 | 45.0 | 96.0 |
| S61 | 0.108 | 0.116 | 0.114 | 0.120 | 0.099 | 0.093 | 0.111 | 0.103 | 0.098 | 0.107 | 45.0 | 85.0 |
| S62 | 0.117 | 0.126 | 0.123 | 0.128 | 0.112 | 0.101 | 0.121 | 0.113 | 0.108 | 0.121 | 45.0 | 76.0 |
| S63 | 0.086 | 0.093 | 0.091 | 0.099 | 0.095 | 0.073 | 0.088 | 0.081 | 0.080 | 0.089 | 50.0 | 123.0 |
| S64 | 0.036 | 0.041 | 0.039 | 0.043 | 0.057 | 0.032 | 0.037 | 0.035 | 0.037 | 0.044 | 50.0 | 113.0 |
| S65 | 0.082 | 0.090 | 0.087 | 0.092 | 0.097 | 0.072 | 0.086 | 0.080 | 0.081 | 0.102 | 50.0 | 96.0 |
| S66 | 0.084 | 0.091 | 0.089 | 0.093 | 0.088 | 0.072 | 0.087 | 0.080 | 0.082 | 0.110 | 50.0 | 86.0 |
| S67 | 0.092 | 0.100 | 0.098 | 0.102 | 0.091 | 0.079 | 0.095 | 0.088 | 0.089 | 0.115 | 52.5 | 58.0 |
| S68 | 0.063 | 0.069 | 0.067 | 0.071 | 0.069 | 0.052 | 0.063 | 0.058 | 0.060 | 0.081 | 55.0 | 128.0 |
| S69 | 0.047 | 0.052 | 0.051 | 0.054 | 0.057 | 0.041 | 0.048 | 0.054 | 0.046 | 0.051 | 55.0 | 116.0 |
| S70 | 0.069 | 0.076 | 0.073 | 0.077 | 0.069 | 0.060 | 0.071 | 0.066 | 0.067 | 0.073 | 55.0 | 86.0 |
| S71 | 0.075 | 0.082 | 0.080 | 0.084 | 0.071 | 0.065 | 0.077 | 0.072 | 0.072 | 0.096 | 55.0 | 86.0 |
| S72 | 0.072 | 0.079 | 0.076 | 0.081 | 0.077 | 0.061 | 0.074 | 0.068 | 0.069 | 0.091 | 55.0 | 76.0 |
| S73 | 0.061 | 0.067 | 0.065 | 0.068 | 0.059 | 0.051 | 0.061 | 0.057 | 0.059 | 0.079 | 55.0 | 66.0 |

Continued

S3 Table. Continued

| S74 | 0.027 | 0.032 | 0.030 | 0.034 | 0.055 | 0.024 | 0.029 | 0.027 | 0.030 | 0.050 | 60.0 | 134.0 |
| --- | --- | --- | --- | --- | --- | --- | --- | --- | --- | --- | --- | --- |
| S75 | 0.034 | 0.039 | 0.037 | 0.041 | 0.053 | 0.029 | 0.035 | 0.032 | 0.035 | 0.043 | 60.0 | 125.0 |
| S76 | 0.045 | 0.050 | 0.048 | 0.051 | 0.047 | 0.039 | 0.045 | 0.043 | 0.045 | 0.056 | 60.0 | 115.0 |
| S77 | 0.049 | 0.055 | 0.053 | 0.056 | 0.057 | 0.042 | 0.050 | 0.047 | 0.049 | 0.060 | 60.0 | 96.0 |
| S78 | 0.063 | 0.068 | 0.067 | 0.070 | 0.053 | 0.053 | 0.063 | 0.059 | 0.060 | 0.067 | 60.0 | 76.0 |
| S79 | 0.020 | 0.025 | 0.023 | 0.027 | 0.057 | 0.019 | 0.023 | 0.022 | 0.025 | 0.047 | 65.0 | 139.0 |
| S80 | 0.020 | 0.022 | 0.022 | 0.023 | 0.020 | 0.018 | 0.021 | 0.020 | 0.021 | 0.023 | 65.0 | 130.0 |
| S81 | 0.026 | 0.029 | 0.028 | 0.031 | 0.041 | 0.024 | 0.027 | 0.026 | 0.028 | 0.032 | 65.0 | 118.0 |
| S82 | 0.029 | 0.033 | 0.031 | 0.034 | 0.039 | 0.026 | 0.029 | 0.028 | 0.030 | 0.039 | 65.0 | 97.5 |
| S83 | 0.021 | 0.025 | 0.023 | 0.026 | 0.048 | 0.019 | 0.022 | 0.021 | 0.023 | 0.040 | 65.0 | 89.0 |
| S84 | 0.194 | 0.201 | 0.200 | 0.204 | 0.147 | 0.157 | 0.194 | 0.179 | 0.141 | 0.151 | 14.2 | 90.2 |
| S85 | 0.175 | 0.188 | 0.185 | 0.193 | 0.209 | 0.149 | 0.182 | 0.169 | 0.142 | 0.167 | 14.5 | 91.5 |
| S86 | 0.180 | 0.189 | 0.187 | 0.193 | 0.127 | 0.143 | 0.181 | 0.165 | 0.134 | 0.146 | 15.0 | 91.0 |
| S87 | 0.199 | 0.206 | 0.205 | 0.206 | 0.135 | 0.163 | 0.200 | 0.185 | 0.143 | 0.144 | 15.8 | 92.8 |
| S88 | 0.187 | 0.193 | 0.192 | 0.193 | 0.119 | 0.152 | 0.186 | 0.172 | 0.145 | 0.155 | 16.3 | 88.7 |
| S89 | 0.187 | 0.194 | 0.193 | 0.199 | 0.138 | 0.151 | 0.187 | 0.172 | 0.148 | 0.169 | 17.7 | 88.7 |
