# Supplementary material for: Coarse woody debris decomposition assessment tool: Model development and sensitivity analysis
Source: PLoS One. 2021 Jun 4;16(6):e0251893. doi: 10.1371/journal.pone.0251893 (PMC8177548; doi:10.1371/journal.pone.0251893)
Supplement: S4 Table — *:D, diameter range, cm; other information, including location and climate conditions are in S5 Table. (DOCX) [file pone.0251893.s004.docx]

S4 Table. Published global decomposition constants for CWD.

| region | Species | Half time (yrs) | References | Note* |
| --- | --- | --- | --- | --- |
| Asia | Pine | 17.3±0.9 | 89 |  |
|  | Oak | 9.9±0.4 | 89 |  |
|  | C. chinensis | 6.5 | 90 |  |
|  | S. superba | 4.7 | 90 |  |
|  | C. concinna | 4.4 | 90 |  |
|  | Tropical rainforest | 0.6-27.7 | 81 |  |
| Europe | Multiple | 4.8-62.4 | 91 |  |
|  | Norway spruce | 14.2-42.0 | 92 |  |
|  | Scots pine | 21.7±13.0 | 80 |  |
|  | Norway spruce | 21.0±9.8 | 80 |  |
|  | Beech | 12.8±9.1 | 80 |  |
|  | Beech | 7.8 | 93 |  |
|  | Spruce | 14.1-26.7 | 84 | 3 species |
|  | Birch | 8.9-23.1 | 84 | 2 species |
|  | Pine | 15.8-46.2 | 84 | 3 species |
|  | Larch | 22.4-46.2 | 84 | 2 species |
|  | Pine | 12.0-69.3 | 88 | D:5-60 cm |
|  | Spruce | 11.7-138.6 | 88 | D:5-60 cm |
|  | Birch | 7.9-17.8 | 88 | D:5-60 cm |
|  | Aspen | 9.8-15.8 | 88 | D:5-60 cm |
|  | Aspen and birch | 10.5±0.3 | 94 |  |
|  | Fir & spruce mean | 21.7±0.7 | 94 |  |
|  | Fir & spruce leaning | 38.5±4.3 | 94 |  |
|  | Fir & spruce fallen | 21.7±0.7 | 94 |  |
|  | Scots pine | 25.7±1.0 | 94 |  |
|  | Siberian pine | 49.5±15.4 | 94 |  |
|  | Alder | 6.8 | 85 |  |
|  | Birch | 12.8 | 85 |  |
|  | Aspen | 9.6 | 85 |  |
|  | Willow | 7.1 | 85 |  |
|  | Ash | 10 | 85 |  |
|  | Scots pine | 17.8 | 85 |  |
| Oceania | Multiple | 1.5-88.9 | 26 | 17 species |
|  | Pine | 5.7-63.0 | 95 |  |
|  | Pine | 9.8 | 86 |  |

* D, diameter range, cm. Continued

Other information, including location and climate conditions are in S4 Table B.

S4 Table. Continued

| Region | Species | Half time (yrs) | References | Note |
| --- | --- | --- | --- | --- |
| North America | Conifers | 13.0-22.0 | 96 | 13 species |
|  | Hardwoods | 8.0-11.0 | 96 | 23 species |
|  | Mixed hardwoods | 8.6-9.2 | 97 | Downed |
|  | Mixed hardwoods | 11.4-13.9 | 97 | Suspended |
|  | Aspen | 8.7 | 98 |  |
|  | Spruce | 9.8 | 98 |  |
|  | Pine | 12.6-16.5 | 98 | 2 species |
|  | Mixed | 3.3 | 99 | flooded |
|  | Mixed | 2.8 | 99 | Unflooded |
|  | Fraser fir | 1.4 | 100 |  |
|  | Red spruce | 11.2 | 100 |  |
|  | Yellow birch | 7.1 | 100 |  |
|  | American beech | 7.2±0.7 | 101 |  |
|  | Sugar maple | 8.8±0.8 | 101 |  |
|  | Yellow birch | 10.7±1.0 | 101 |  |
|  | Red spruce | 23.9 | 102 |  |
|  | Balsam fir | 21 | 102 |  |
|  | Pine | 99.0±30.8 | 103 |  |
|  | Hemlock | 19.3±4.5 | 103 |  |
|  | Red alder | 1.3-19.8 | 104 | D:1-12 cm |
|  | Douglas fir | 3.4-115.5 | 104 |  |
|  | Mixed | 8.4 | 105 |  |
|  | Pine | 4.4 | 106 |  |
|  | Aspen | 25.3±0.6 | 107 | Snag |
|  | Jack pine | 45.6±1.5 | 107 | Snag |
|  | Balsam fir | 56.4±2.8 | 107 | Snag |
|  | Black spruce | 119.5±6.2 | 107 | Snag |
|  | Black spruce | 9.9±8.4 | 83 |  |
|  | Conifer | 9.8-198.0 | 108 |  |
|  | Conifer | 13.9-115.5 | 25 |  |
|  | Deciduous | 7.2-28.9 | 25 |  |
|  | Mixed | 8.7-33.0 | 25 |  |
|  | Multiple | 2.1-88.9 | 34 | 48 species |
|  | Multiple | 4.0-230.0 | 2 |  |
|  | Mixed deciduous | 8.2±17.4 | 109 |  |
| South America | Tropical evergreen | 4.2 | 25 |  |
|  | Mixed | 1.1-5.8±9.0 | 82 | D: >10 cm |
|  | Multiple | 0.6-14.0 | 87 | 26 species |
| This study | Mixed | 3.4-67.3 | This study | D:6-45 cm |
