# Supplementary material for: Coarse woody debris decomposition assessment tool: Model development and sensitivity analysis
Source: PLoS One. 2021 Jun 4;16(6):e0251893. doi: 10.1371/journal.pone.0251893 (PMC8177548; doi:10.1371/journal.pone.0251893)
Supplement: S5 Table — *: DM, decay model; SE, single exponent; NS, Northern and Southern Germany; LR, Leningrad Region; ML, multiple locations. The order of lines is the same as that in S4 Table. (DOCX) [file pone.0251893.s005.docx]

S5 Table. Locations and climate for the published global decomposition constants of CWD*.

| Region | Latitude | Longitude | Elevation  (m) | Temperature (°C) | Precipitation  (mm) | DM |
| --- | --- | --- | --- | --- | --- | --- |
| Asia | 33.43° N | 108.45° E | 1524-1585 | 8.5-12.5 | 750-1150 | SE |
|  | 33.43° N | 108.43° E | 1597-1685 | 8.5-12.5 | 750-1150 | SE |
|  | 23.17° N | 112.5° E | 14-1000 | 21.5 | 1956 | SE |
|  | 23.17° N | 112.5° E | 14-1001 | 21.5 | 1956 | SE |
|  | 23.17° N | 112.5° E | 14-1002 | 21.5 | 1956 | SE |
|  | 1.38° N | 110.32° E |  | 26.4 | 4581 | SE |
| Europe | 52.4°-53.13° N | 7.27°-7.45° W |  | 9.3 | 850 |  |
|  | 59.57°-60.17° N | 8.97°-10.92° E | 60-580 | -8.2 to -4.7 | 675-820 | SE |
|  | NS Germany | NS Germany | 70-950 | 7--11 | 645-1700 | SE |
|  | NS Germany | NS Germany | 70-950 | 7--11 | 645-1700 | SE |
|  | NS Germany | NS Germany | 70-950 | 7--11 | 645-1700 | SE |
|  | Central Germany | Central Germany | 500 | 7 | 1032 | SE |
|  | 52°, 56°, 59° N | 104°, 93°, 31°-32° E |  | -0.6; -3.2; 3.4 | 216; 361; 708 | SE |
|  | 49°-59 °N | 140°, 104°, 93° E, 31°-32° E |  | -1.9; -0.6; -3.2; 3.3 | 909; 216; 361; 707 | SE |
|  | 49°-59 °N | 140°, 104°, 93° E, 31°-32° E |  | -1.9; -0.6; -3.2; 3.4 | 909; 216; 361; 708 | SE |
|  | 49°, 52°N, 56°N | 140°; 104°; 93° E |  | -1.9; -0.6; -3.2 | 909; 216; 361 | SE |
|  | LR, Russia | LR, Russia |  | 3--5 | 500--800 | SE |
|  | LR, Russia | LR, Russia |  | 3--5 | 500--800 | SE |
|  | LR, Russia | LR, Russia |  | 3--5 | 500--800 | SE |
|  | LR, Russia | LR, Russia |  | 3--5 | 500--800 | SE |
|  | ML, Russia | ML, Russia |  | -3.0 to 3.6 | 600--750 | SE |
|  | ML, Russia | ML, Russia |  | -3.0 to 3.6 | 600--750 | SE |
|  | ML, Russia | ML, Russia |  | -3.0 to 3.6 | 600--750 | SE |
|  | ML, Russia | ML, Russia |  | -3.0 to 3.6 | 600--750 | SE |
|  | ML, Russia | ML, Russia |  | -3.0 to 3.6 | 600--750 | SE |
|  | ML, Russia | ML, Russia |  | -3.0 to 3.6 | 600--750 | SE |
|  | 68.35° N | 18.82° E | 350--400 | -39.0--21.3 | 352 |  |
|  | 68.35° N | 18.82° E | 350--400 | -39.0--21.3 | 352 |  |
|  | 68.35° N | 18.82° E | 350--400 | -39.0--21.3 | 352 |  |
|  | 68.35° N | 18.82° E | 350--400 | -39.0--21.3 | 352 |  |
|  | 68.35° N | 18.82° E | 350--400 | -39.0--21.3 | 352 |  |
|  | 68.35° N | 18.82° E | 350--400 | -39.0--21.3 | 352 |  |
| Oceania | country-wide | country-wide |  |  |  |  |
|  | 35.33° S | 149.33° E | 720 |  | 622 | SE |
|  | 43.42°S | 172.27° E |  |  | 870 | SE |

*DM, decay model; SE, single exponent; NS, Northern and Southern Germany; Continued

LR, Leningrad Region; ML, multiple locations. The order of lines is the same as that in S4 Table A.

S5 Table. Continued*

| Region | Latitude | Longitude | Elevation  (m) | Temperature  (°C) | Precipitation  (mm) | DM |
| --- | --- | --- | --- | --- | --- | --- |
| North America | LA-MN, USA | MN-ME, USA |  | 1.4--19.8 | 550-2010 |  |
|  | LA-MN, USA | MN-ME, USA |  | 1.4--19.8 | 550-2010 |  |
|  | LA, USA | LA, USA |  |  |  |  |
|  | LA, USA | LA, USA |  |  |  |  |
|  | MN, USA | MN, USA |  |  | 639 | SE |
|  | MN, USA | MN, USA |  |  | 639 | SE |
|  | MN, USA | MN, USA |  |  | 639 | SE |
|  | MS, USA | MS, USA |  |  |  | SE |
|  | MS, USA | MS, USA |  |  |  | SE |
|  | 35.57° N | 83.48° W | 1676-1921 |  |  | SE |
|  | 35.57° N | 83.48° W | 1676-1921 |  |  | SE |
|  | 35.57° N | 83.48° W | 1676-1921 |  |  | SE |
|  | 43.93° N | 71.75° W |  | 5.5 |  | SE |
|  | 43.93° N | 71.75° W |  | 5.5 |  | SE |
|  | 43.93° N | 71.75° W |  | 5.5 |  | SE |
|  | 44° N | 71° W | 540-880 | 5.4-2.3 | 1250-1420 |  |
|  | 44° N | 71° W | 540-880 | 5.4-2.3 | 1250-1420 |  |
|  | WA, USA | WA, USA | 360-900 |  |  | SE |
|  | WA, USA | WA, USA | 360-900 |  |  | SE |
|  | WA, USA | WA, USA |  | 9.7 |  | SE |
|  | WA, USA | WA, USA |  | 9.7 |  |  |
|  | 35.07° N | 83.42° W | 730-990 | 12.6 | 1820 |  |
|  | FL, USA | FL, USA |  |  |  | SE |
|  | 48.43°-48.48° N | 79.43°-79.3° W |  | 0.7 | 889.8 | L&E |
|  | 48.43°-48.48° N | 79.43°-79.3° W |  | 0.7 | 889.8 | L&E |
|  | 48.43°-48.48° N | 79.43°-79.3° W |  | 0.7 | 889.8 | L&E |
|  | 49.42°-49.83° N | 79.3°-78.68° W |  |  |  | L&E |
|  | 55.88° N | 98.33° W |  | 0.8 | 438.5 |  |
|  | MC | MC |  |  |  | SE |
|  | MC | MC |  |  |  | SE |
|  | MC | MC |  |  |  | SE |
|  | MC | MC |  |  |  | SE |
|  | LA, USA - AB, CA | OR, USA - NB, CA | 9-1370 | -4.8 to 19.5 | 280-3300 |  |
|  | Temperate Forests | Temperate Forests |  |  |  |  |
|  | 20° N | 88° W |  | 25 | 1100 |  |
| South America | 2.5° S | 60° W |  | 26.7 | 2200 | SE |
|  | 1.6° N | 61.22° W |  |  | 1750-2000 | SE |
|  | 5.3° N | 52.92° W |  |  | 3041 | SE |
| This study | 14.2°-65.0° N | 66°-139° W | 337546 | -11.8 to 26.5 | 181.3-6142.8 | PE |

** FL, LA, ME, MN, MS, WA are states Florida, Louisiana, Maine, Minnesota, Mississippi, Washington, USA; MC stands for multiple countries; PE stands for Power-Exponent.
