# Supplementary figures and images for: Coarse woody debris decomposition assessment tool: Model development and sensitivity analysis
Source: PLoS One. 2021 Jun 4;16(6):e0251893. doi: 10.1371/journal.pone.0251893 (PMC8177548; doi:10.1371/journal.pone.0251893)

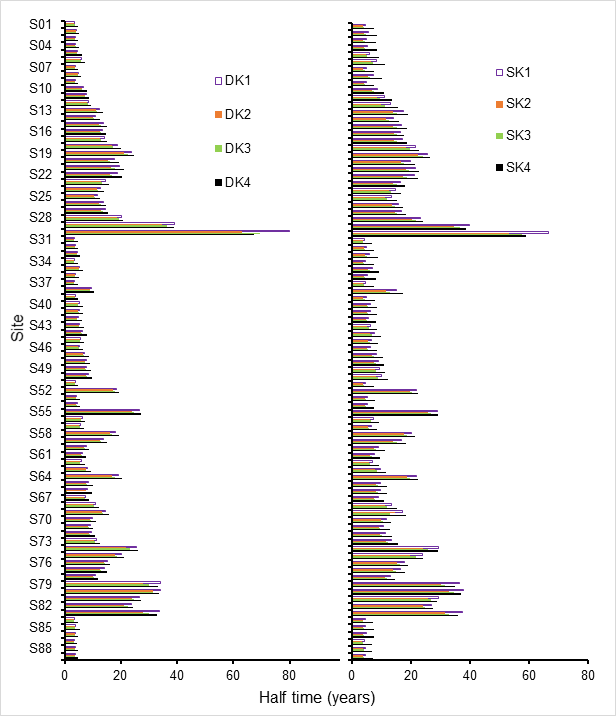

Supplement: S1 Fig — DK1 –DK3 (left) are calculated for downed deadwood decay using k1, k2 and k3 from Eqs 21–23, respectively. DK4 (left) is calculated for the downed deadwood using k4 and k5 from the Eq 24. SK1 –SK4 (right) are for standing deadwood calculated using equations as described above for downed deadwood. (TIF) [file pone.0251893.s006.tif]

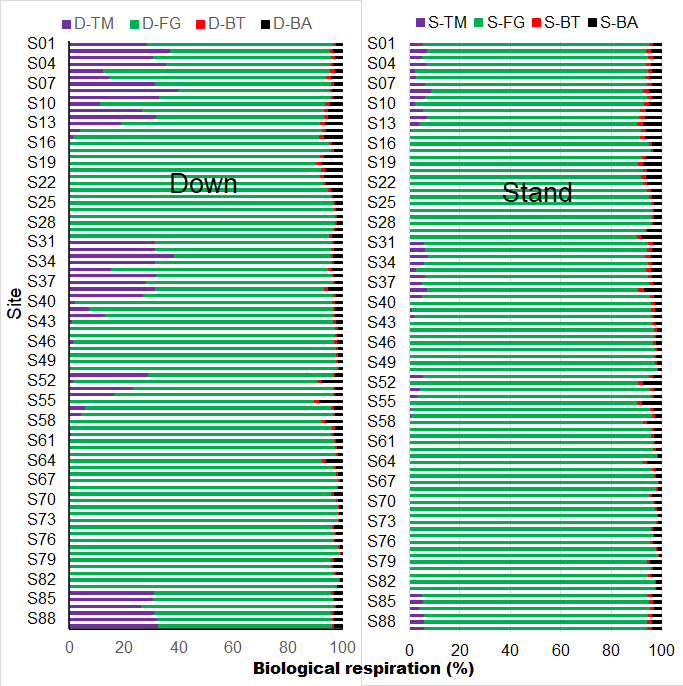

Supplement: S2 Fig — The figure on the left for downed deadwood and that on the right is for standing deadwood. (TIF) [file pone.0251893.s007.tif]
