# Supplementary material for: Coarse woody debris decomposition assessment tool: Model development and sensitivity analysis
Source: PLoS One. 2021 Jun 4;16(6):e0251893. doi: 10.1371/journal.pone.0251893 (PMC8177548; doi:10.1371/journal.pone.0251893)
Supplement: Attachment — Submitted filename: Responses_To_Editor_Reviewers-MS1.docx [file pone.0251893.s008.docx]

Journal Requirements:

When submitting your revision, we need you to address these additional requirements.

1. Please ensure that your manuscript meets PLOS ONE's style requirements, including those for file naming. The PLOS ONE style templates can be found at

https://journals.plos.org/plosone/s/file?id=wjVg/PLOSOne_formatting_sample_main_body.pdf and <https://journals.plos.org/plosone/s/file?id=ba62/PLOSOne_formatting_sample_title_authors_affiliations.pdf>

**Response: Thanks. While revising we have taken care to ensure that PLOS ONE’s style guidelines have been followed.**

2.In your Data Availability statement, you have not specified where the minimal data set underlying the results described in your manuscript can be found. PLOS defines a study's minimal data set as the underlying data used to reach the conclusions drawn in the manuscript and any additional data required to replicate the reported study findings in their entirety. All PLOS journals require that the minimal data set be made fully available. For more information about our data policy, please see http://journals.plos.org/plosone/s/data-availability.

Upon re-submitting your revised manuscript, please upload your study’s minimal underlying data set as either Supporting Information files or to a stable, public repository and include the relevant URLs, DOIs, or accession numbers within your revised cover letter. For a list of acceptable repositories, please see http://journals.plos.org/plosone/s/data-availability#loc-recommended-repositories. Any potentially identifying patient information must be fully anonymized.

**Response: The main data used to parameterize the model were obtained from Daymet database (see reference: Thornton et al, 2016). The coordinates of the eighty-nine sites for obtaining climate data to analyze the model sensitivity are in S1 Table. S1 Table, S2 Table, and S4 Table B have been added to let readers and model users can see the data we used, enabling them to also test the model using those data. The climate, coordinates and altitude of corresponding locations were from Daymet database used to analyze the model sensitivity, as long as the data are downloaded from Daymet and used to parameterize the model, the results from the model outputs can be used to compare with the results shown in this manuscript. The data in Table 1, S1 Table and S2 Table are needed if anyone who wants to test this model. Other data used to parameterize the model are in this manuscript. S4 Table A and S4 Table B are those data collected for the comparison of modeling results and those from other studies. S3 Table and S4 Table A in this version are the Table 3 and Table 4 in previous version. Additionally, S1 Fig. and S2 Fig. in this version are the Fig. 4 and Fig 21 in previous version.**

Important: If there are ethical or legal restrictions to sharing your data publicly, please explain these restrictions in detail. Please see our guidelines for more information on what we consider unacceptable restrictions to publicly sharing data: http://journals.plos.org/plosone/s/data-availability#loc-unacceptable-data-access-restrictions. Note that it is not acceptable for the authors to be the sole named individuals responsible for ensuring data access.

**Response: There are no restrictions to data usage.**

We will update your Data Availability statement to reflect the information you provide in your cover letter.

**Response: Thanks.**

3. PLOS requires an ORCID iD for the corresponding author in Editorial Manager on papers submitted after December 6th, 2016. Please ensure that you have an ORCID iD and that it is validated in Editorial Manager. To do this, go to ‘Update my Information’ (in the upper left-hand corner of the main menu), and click on the Fetch/Validate link next to the ORCID field. This will take you to the ORCID site and allow you to create a new iD or authenticate a pre-existing iD in Editorial Manager. Please see the following video for instructions on linking an ORCID iD to your Editorial Manager account: https://www.youtube.com/watch?v=_xcclfuvtxQ

**Response: Yes, my ORCID is 0000-0002-0941-8345and it has been validated in Editorial Manager.**

4.Thank you for stating the following in the Acknowledgments Section of your manuscript:

"Funding to install the FWDE was provided by the USDA Forest Service and Michigan

Technological Univ. Current work is being supported by the U.S Dept. of Energy (DE764

SC0016235), US NSF (DEB 1754603) and the USDA Forest Service."

We note that you have provided funding information that is not currently declared in your Funding Statement. However, funding information should not appear in the Acknowledgments section or other areas of your manuscript. We will only publish funding information present in the Funding Statement section of the online submission form.

Please remove any funding-related text from the manuscript and let us know how you would like to update your Funding Statement. Currently, your Funding Statement reads as follows:

"The funding sources have been listed in Acknowledgments."

Please include your amended statements within your cover letter; we will change the online submission form on your behalf.

**Response: Thank you for the kind reminder. The funding information in Acknowledgement has been deleted.**

5. Thank you for stating the following in your Competing Interests section:

"NO"

Please complete your Competing Interests on the online submission form to state any Competing Interests. If you have no competing interests, please state "The authors have declared that no competing interests exist.", as detailed online in our guide for authors at http://journals.plos.org/plosone/s/submit-now

This information should be included in your cover letter; we will change the online submission form on your behalf.

Please know it is PLOS ONE policy for corresponding authors to declare, on behalf of all authors, all potential competing interests for the purposes of transparency. PLOS defines a competing interest as anything that interferes with, or could reasonably be perceived as interfering with, the full and objective presentation, peer review, editorial decision-making, or publication of research or non-research articles submitted to one of the journals. Competing interests can be financial or non-financial, professional, or personal. Competing interests can arise in relationship to an organization or another person. Please follow this link to our website for more details on competing interests: http://journals.plos.org/plosone/s/competing-interests

**Response: Thank you for your help. The authors have declared that no competing interests exist.**

6.We note that Figure(s) 2 in your submission contain map images which may be copyrighted. All PLOS content is published under the Creative Commons Attribution License (CC BY 4.0), which means that the manuscript, images, and Supporting Information files will be freely available online, and any third party is permitted to access, download, copy, distribute, and use these materials in any way, even commercially, with proper attribution. For these reasons, we cannot publish previously copyrighted maps or satellite images created using proprietary data, such as Google software (Google Maps, Street View, and Earth). For more information, see our copyright guidelines: http://journals.plos.org/plosone/s/licenses-and-copyright.

We require you to either (1) present written permission from the copyright holder to publish these figures specifically under the CC BY 4.0 license, or (2) remove the figures from your submission:

a) You may seek permission from the original copyright holder of Figure(s) 2 to publish the content specifically under the CC BY 4.0 license.

We recommend that you contact the original copyright holder with the Content Permission Form (http://journals.plos.org/plosone/s/file?id=7c09/content-permission-form.pdf) and the following text:

“I request permission for the open-access journal PLOS ONE to publish XXX under the Creative Commons Attribution License (CCAL) CC BY 4.0 (http://creativecommons.org/licenses/by/4.0/). Please be aware that this license allows unrestricted use and distribution, even commercially, by third parties. Please reply and provide explicit written permission to publish XXX under a CC BY license and complete the attached form.”

Please upload the completed Content Permission Form or other proof of granted permissions as an "Other" file with your submission.

In the figure caption of the copyrighted figure, please include the following text: “Reprinted from [ref] under a CC BY license, with permission from [name of publisher], original copyright [original copyright year].”

b)	If you are unable to obtain permission from the original copyright holder to publish these figures under the CC BY 4.0 license or if the copyright holder’s requirements are incompatible with the CC BY 4.0 license, please either i) remove the figure or ii) supply a replacement figure that complies with the CC BY 4.0 license. Please check copyright information on all replacement figures and update the figure caption with source information. If applicable, please specify in the figure caption text when a figure is similar but not identical to the original image and is therefore for illustrative purposes only.

The following resources for replacing copyrighted map figures may be helpful:

USGS National Map Viewer (public domain): http://viewer.nationalmap.gov/viewer/

The Gateway to Astronaut Photography of Earth (public domain): http://eol.jsc.nasa.gov/sseop/clickmap/

Maps at the CIA (public domain): https://www.cia.gov/library/publications/the-world-factbook/index.html and https://www.cia.gov/library/publications/cia-maps-publications/index.html

NASA Earth Observatory (public domain): http://earthobservatory.nasa.gov/

Landsat: http://landsat.visibleearth.nasa.gov/

USGS EROS (Earth Resources Observatory and Science (EROS) Center) (public domain): http://eros.usgs.gov/#

Natural Earth (public domain): http://www.naturalearthdata.com/

**Response: Figure 2 (current Fig 1) was replaced using the data from Natural Earth (please read the caption). We also added a table to show the exact locations of sites for which climate data was used for the model sensitivity analysis. We also have added S2 Table as a data supplement so readers can see the differences in climate among the sites used. Climate data was obtained from Daymet database (Thornton et al., 2016 in References). If anyone wishes to test this model, they can download that same data there using the coordinates in S1 Table to parameterize the model.**

Additional Editor Comments:

I now have one report from an expert reviewer who considers this study interesting, but also raises several technique concerns, including logic in the introduction and some issues related to modeling. My decision is Major revision.

**Response: Thank you. We have made changes, described below, which we feel address the reviewer’s comments.**

[Note: HTML markup is below. Please do not edit.]

Reviewers' comments:

Reviewer's Responses to Questions

Comments to the Author

1. Is the manuscript technically sound, and do the data support the conclusions?

The manuscript must describe a technically sound piece of scientific research with data that supports the conclusions. Experiments must have been conducted rigorously, with appropriate controls, replication, and sample sizes. The conclusions must be drawn appropriately based on the data presented.

Reviewer #1: Partly

**Response: Thanks. To confirm the model reliability, we used eighty-nine datasets to test the model across a wide climatic range from tropical to boreal zones and with a large difference in elevation (3 to 2,824 m above mean sea level).**

**This manuscript is only one part of the model development, which is mainly used to introduce the modeling methods and the results from model testing for determining whether or not the model is in line with the design objectives. This is especially important for a new model. Accordingly, we used a large dataset covering a wide geographic range from tropical to boreal zones. We will also improve the quality of this manuscript based on all of your comments**.

2. Has the statistical analysis been performed appropriately and rigorously?

Reviewer #1: Yes

**Response: Thanks.**

3. Have the authors made all data underlying the findings in their manuscript fully available?

The PLOS Data policy requires authors to make all data underlying the findings described in their manuscript fully available without restriction, with rare exception (please refer to the Data Availability Statement in the manuscript PDF file). The data should be provided as part of the manuscript or its supporting information, or deposited to a public repository. For example, in addition to summary statistics, the data points behind means, medians and variance measures should be available. If there are restrictions on publicly sharing data—e.g. participant privacy or use of data from a third party—those must be specified.

Reviewer #1: No

**Response: This is similar to the second Journal requirement. Please see the response there. We also added additional information to the captions of figures and tables. S1 Table, S2 Table and S4 Table B was added as supplements with the data used for the model sensitivity analysis.**

4. Is the manuscript presented in an intelligible fashion and written in standard English?

PLOS ONE does not copyedit accepted manuscripts, so the language in submitted articles must be clear, correct, and unambiguous. Any typographical or grammatical errors should be corrected at revision, so please note any specific errors here.

Reviewer #1: No

**Response: During revision, we have carefully edited the manuscript to clarify language, with multiple co-authors checking for typographical or grammatical errors.**

5. Review Comments to the Author

Please use the space provided to explain your answers to the questions above. You may also include additional comments for the author, including concerns about dual publication, research ethics, or publication ethics. (Please upload your review as an attachment if it exceeds 20,000 characters)

Reviewer #1: This is potentially interesting study dealing with modelling yet poorly studied decomposition dynamics of coarse woody debris with qualifying factors influencing wood decomposition rate.

However, the manuscript needs major revisions.

The introduction would be more logical if the text would be focused on the factors influencing the decomposition rates and the mechanisms of this influence. The essence of the developed model should be better explained. Moreover, the objective of this study should be clearly stated.

**Response: We have revised the introduction to address this, lines 46 to 86 have been revised or reworded (please see the tracking version). The essence of the model is explained on lines 90 to 96, and the objectives of this paper is now clearly stated on lines 97 to 102.**

The importance of processes leading to mass loss of CWD, such as fragmentation, can be stated in the introduction, not in the methods section. The same concerns bacteria and other biological agents. It is stated that the contribution of bacteria to overall deadwood decomposition is small, but later the equation including some figure for the mass of bacteria is presented. How this mass was estimated?

I did not understand, where the coefficients used in the separate model equations came from?

**Response: Because this model is process-based, we would like to leave processes in methods. We hope those processes can be verified by users. Therefore, we did not want to use only one complicated equation to describe CWD decay, and every process can be tested independently by users.**

**Most biological coefficients were estimated based on the** **biological characteristics. For example, fungal growth is based on their survival conditions, and we assumed that the max value was 1 when all conditions were optimal for the fungi, the min value was 0 when the environmental conditions did not support fungal life. Accordingly, there are no constant coefficients for all decomposers. In order to be clarify, we revised line 147, and added sentences to the end of this paragraph (new lines 153-158) containing an equation used to calculate fungal activity in the model. Mass of each decomposer was estimated based on their activity depending on their biological characteristics and surviving conditions that are the model inputs (lines 148 to 158 for fungi, lines 172 to 173 for termites, line 195 for beetles).**

Please, explain, how did you estimate e.g. fungal and beetle biomass, or the daily CWD consumed by termites. This information supported by references can be provided e.g. in supplements. It is impossible to evaluate the results of the sensitivity analysis without understanding model inputs. Moreover, the comparisons of decay rates calculated in different ways based on suggested model are of restricted value for potential users, because it is unclear, how where the decay constants calculated?

**Response: The biological biomass was estimated based on their activity assessed on their surviving conditions (mentioned above), including temperature, moisture and food source, which should be described by the relative equations and corresponding text. Termite consumption coefficients were from literatures that have been referred. Decay constant is only averaged from daily decomposition because this is a process-based model. The decomposers’ activities are altered as temperature and moisture changed, and the time steps for biological consumption is hourly such that the climate data used for the model input are daily minimum and maximum temperature and daily precipitation are needed (see line 279). The Figure 19 showed that annual decomposition was not constant, the decomposition day-to-day or month-to-month were changed as environmental conditions changed. If decay constants were used to calculate the decay rates, the models were empirical, not process-based.**

The next question is: how the environmental variables were included in the model? This is especially important as the main result from the sensitivity analysis is that deadwood decomposition was influenced by the main inputs, temperature, precipitation, snow, geographical location and geomorphic altitude, as well as wood properties such as density and size. How were these variables used as model inputs?

**Response: Sorry for the missing explanations. Wording has been added to Sensitivity Analysis section to clarify what the inputs are (see lines 276 to 280). We added text to clarify what the main inputs are: the size, species and mass of CWD, soil, vegetation, and climate data [lines 277 to 280 for main inputs; lines 299 to 300, 310 to 313 for size and wood density used for the sensitivity analysis (Table 1 and Table 2)]. Climate data included daily minimum and maximum temperature, daily precipitation (rainfall and snowfall), and day PAR (Photosynthetically active radiation) (see lines 279 to 280 of the revised manuscript).**

Conclusions should not repeat results!

**Response: We revised this section, lines 722 to 736 and line 743 to 748 were new text, other lines were reworded.**

The number of tables and figures can be shortened, some of them can be moved to supplements.

**Response: Original Table 3 and Figure 3 and Figure 21 have been moved into the supplements section as S3 Table, S1 Fig and S2 Fig.**

The terminology used should be consistent. For example, the terms coarse woody debris, woody debris, deadwood, dead wood are used throughout the text.

**Response: Thanks. We have strived to be more consistent during revision, and now “coarse woody debris” and the acronym (CWD) are the primary terms used.**

Fig. 1 could be better developed by adding e.g. references to the equations used, explaining, what the arrows and question marks mean etc. Fig. 1 contains mistakes: leaching

**Response： Text has been added to the Fig. 1 caption to explain the meaning of arrows and the misspelling has been corrected.**

Here are some specific comments on the Introduction and methods showing that the text should be revised.

Abstract.

Lines 20-22. It would be better to use the same order: either from tropical to boreal or the other way round in listing temperature, precipitation and other ranges.

**Response: Ranges for temperature, precipitation and snowfall are now arranged from tropical to boreal. We cannot do so for elevation as it is not related to climatic zone.**

Line 22. Annual snowfall?

**Response: Thanks. The correction as made.**

The terms ‘decomposition’ and ‘decay’ should be defined and should not be used as synonyms.

**Response: Decomposition was used now.**

Introduction

Line 42. Habitats

**Response: The suggested change has been made.**

Line 43. Potential risk of wildfires

**Response: The suggested change has been made.**

Line 48. Remove the word ‘natural’.

**Response: This has been done.**

Lines 55-56. The statement needs a reference.

**Response: A reference was added.**

Lines 61-62. ‘Rots’ do not ‘decompose’. (wording)

**Response: Yes. The word “fungi” has been added as needed to correct the statements (i..e. ..soft-rot fungi can decompose…).**

Lines 67-71. This is not true. There is a huge number of studies on the decomposition of CWD! The whole paragraph should be re-written.

**Response: This has been reworded. The paragraph was not meant to state how many studies on CWD decay exist, it described only some differences in roles among fungi (lines 58 to 63 of the revised version).**

Line 73. What is ‘wood location’?

**Response: Sorry for the non-specific term used. “location” meant “position”. In the revised manuscript we have replaced location with “position (standing vs. downing)”, to make this clear.**

Lines 76-77. What is ‘mixed with linear and exponential models’?

**Response: This has been reworded to state “more complex models”**

Lines 83-90. The point here is unclear. The essence of the models should be better described.

**Response: We have reworded the sentence to read “**There are several computer models used to estimate CWD decomposition. For example, Yin (1999) developed a computer model based on the methodology suggested by Agren and Bosatta (1987) to analyze C and nitrogen dynamics in forest soils. Yasso, a process-based soil C model with a woody litter decomposition subroutine, was used to assess woody litter decomposition, and the results were consistent with the litterbag data from Canada (Liski et al., 2005; Palosuo et al., 2005). On the basis of these two computer models, Zell et al. (2009) developed a computer model used to reanalyze woody debris decomposition using the same data as Yin (1999) used. Although these computer models are not fully process-based models, they give better estimations of CWD decomposition than the simple empirical decomposition models. **” to help clarify the point being made.**

Methods

Line 109. The sentence is very unclear. The information from ‘the literature on CWD decomposition’ that was used in the model should be specified.

**Response: We have reviewed over two hundred literature sources in developing CWDDAT. Most of literatures we have were involved in this model development. The citations given in this sentence were the most important. The sentence has been somewhat revised, and now reads “**The CWDDAT model was developed from coarse woody debris decomposition studies in the literature ()**”**

Line 177. Beetles are not wood decomposing

**Response: A literature (Ulyshen, 2014) was added for the decomposers.** **Added sentence: “Although many beetles cannot directly consume CWD, they can play a role in fragmentation, helping reduce CWD mass, and then those fragments can be decomposed by fungi and bacteria.” (see lines 185 to 187)**

Line 304. The ‘decay constants’ cannot be ‘obtained from methods’.

**Response: This has been changed and now refers to decomposition constants obtained from different models.**

6. PLOS authors have the option to publish the peer review history of their article (what does this mean?). If published, this will include your full peer review and any attached files.

If you choose “no”, your identity will remain anonymous but your review may still be made public.

Do you want your identity to be public for this peer review? For information about this choice, including consent withdrawal, please see our Privacy Policy.

Reviewer #1: Yes: Ekaterina Shorohova

[NOTE: If reviewer comments were submitted as an attachment file, they will be attached to this email and accessible via the submission site. Please log into your account, locate the manuscript record, and check for the action link "View Attachments". If this link does not appear, there are no attachment files.]

While revising your submission, please upload your figure files to the Preflight Analysis and Conversion Engine (PACE) digital diagnostic tool, https://pacev2.apexcovantage.com/. PACE helps ensure that figures meet PLOS requirements. To use PACE, you must first register as a user. Registration is free. Then, login and navigate to the UPLOAD tab, where you will find detailed instructions on how to use the tool. If you encounter any issues or have any questions when using PACE, please email PLOS at figures@plos.org. Please note that Supporting Information files do not need this step.

**Response: Yes. We have done as you suggested. All figures have been uploaded to PACE for the analysis and correction. The figures in current version of manuscript are downloaded from PACE.**
